# The Supercapsular Percutaneously Assisted Total Hip (SuperPATH) Approach Revisited: Technique Improvements after the Perioperative Experience of 344 Cases

**DOI:** 10.3390/life12070981

**Published:** 2022-06-30

**Authors:** Dimitrios A. Flevas, Georgios A. Tsakotos, Leonardos N. Benakis, Grigorios G. Sasalos, Anastasios V. Tokis

**Affiliations:** 1 Arthroscopy and Orthopaedic Surgery Department, Metropolitan Hospital, Neo Faliro, 185 47 Athens, Greece; gtsakotos@gmail.com (G.A.T.); lbenakis@gmail.com (L.N.B.); gregorysasalos@gmail.com (G.G.S.); a.tokis@arthroscopycenter.gr (A.V.T.); 2 Department of Anatomy, School of Medicine, National and Kapodistrian University of Athens, 157 72 Athens, Greece

**Keywords:** SuperPATH approach, hip replacement, hip reconstruction, minimal invasive hip, hip arthroplasty

## Abstract

The SuperPATH approach is a direct superior portal-assisted approach for total hip arthroplasty (THA) that utilizes the interval between the gluteus minimus and the piriformis to access the hip capsule. Patients and Methods: SuperPATH arthroplasty was performed by a single surgeon between December of 2016 and December of 2021 in 344 cases. The technique described by Chow was performed in all cases. The average length of stay was 1.3 days, and all patients were discharged for home. All patients were mobilized on the day of the operation. Six cases presented complications: four intraoperative femur fractures and two peroneal nerve palsies. No infection and no hip dislocation were noticed in any case. Modifications: We recommend that the patient be placed as far from the surgeon as possible as well as the use of a sterilized standard linen pack to elevate the foot and internally rotate the hip. In addition, regarding instrumentation, we recommend the use of a 4.5 mm drill for the first guidance femur drilling and a standard corkscrew for femoral head removal. Finally, we propose a different reduction technique using a hook. The use of the SuperPATH approach allows for maximal tissue sparing through preservation of external rotators and minimizing stretching of the gluteus medius. There is no range of motion restrictions postoperatively and patients can achieve a high level of function with a very low dislocation risk and reduced inpatient stay. Furthermore, an incision extension is possible if needed in complex cases. For surgeons familiar with the standard posterolateral approach, the SuperPATH approach is a reliable and safe method with promising results for the patient. In order to improve the surgical effect and facilitate some steps in the procedure, we share our experience and recommend some modifications.

## 1. Introduction

An effective method to manage hip osteoarthritis has been total hip arthroplasty (THA). THA, one of the most reproducible and frequently performed orthopedic surgical procedures, also provides effective treatment for disorders such as femoral neck fracture, femoral head necrosis, and acetabular dysplasia [1,2]. This operation has increasingly gained popularity amongst surgeons, because it relieves pain effectively and is associated with early mobilization and improved life quality among patients [3]. There are three main classical approaches to the hip for THA: posterolateral, anterolateral, and anterior. There have been a plethora of large clinical studies that have shown excellent results from all these main classical methods of THA [4]. However, issues regarding pain control, length of postoperative rehabilitation, and complications from the surgical approach resulted in modifications that improved those traditional approaches to become more tissue friendly [2].

The alterations that were adopted led to minimally invasive surgical (MIS) approaches for elective THA that became popular due to the potential for decreased muscular damage, pain, blood loss, and time to mobilization [5]. MIS approaches represent modifications of the conventional approaches with an incision length less than 10 cm, resulting in lower tissue traumatization. The approaches available are divided into two groups: muscle-sparing and mini-incision approaches [6]. More commonly used modifications currently include the direct anterior approach (DAA), the anterior supine intermuscular approach (ASI or OCM), and the modified mini-posterior approach. Each of these MIS approaches has been shown to be safe and effective when the period of its “learning curve” is over [2]. This learning curve period has been reported to differ based on approach, author, and patient population [7,8]. However, in order to further reduce tissue trauma, some additional modifications to MIS approaches have been applied. One such modified posterolateral approach, the supercapsular percutaneously assisted total hip (SuperPATH) approach, has been introduced rather recently [9].

The supercapsular percutaneously assisted total hip (SuperPATH^®^, MicroPort Orthopedics Inc., Arlington, TN, USA) belongs to the group of MIS posterior approaches and shares some common ground with the traditional posterior approach. It was presented by Chow et al. in 2011 and consisted a combination of the percutaneously-assisted total hip (“PATH”, Wright Medical Technology, Inc., Memphis, TN, USA) approach [10] and the supercapsular (“SuperCap”, Wright Medical Technology, Inc., Memphis, TN, USA) approach [11]. The SuperPATH approach adopts useful elements of both procedures [9]. The interval between the gluteus minimus and piriformis is used for the hip to be approached by retracting both the gluteus medius and gluteus minimus anteriorly. Thus, the short external rotator muscles and iliotibial band are not violated, and the hip is not dislocated. Furthermore, an accessory percutaneous portal is used to prepare the acetabulum as it is performed in the PATH approach [10]. These characteristics theoretically reduce the risk of postoperative dislocation and allow for decreased time to ambulation, length of stay, 30 day readmission rates, in-hospital costs, and blood loss [5,12].

The purpose of this paper was to report our perioperative results with the use of the SuperPATH approach and share our experience with this technique. Moreover, by proposing some modifications in crucial steps of the procedure, we aim to make this approach more friendly to surgeons that already use it. The ultimate goal is to optimize the approach and its surgical results.

## 2. Patients and Methods

The technique for the SuperPATH approach has been thoroughly described by Chow, and its major steps have been analyzed [13]. In our orthopedic department, we have used the SuperPATH approach for total hip replacements and hemiarthroplasties in femoral neck fractures since December 2016. From December 2016 to December 2021, we have performed the SuperPATH approach on 335 patients. In nine patients (six females and three males) we performed bilateral total hip arthroplasty using the SuperPATH approach. Subsequently, we have performed the approach on 344 hips. All procedures have been performed by the senior author (A.V.T.) as the primary surgeon. We have used the technique for the SuperPATH approach described by Chow.

The patients were 215 females and 120 males. The mean age was 72 ± 4 years for females and 77 ± 3 for males. Eighty-eight patients (25%) were obese: 58 females and 30 males. Twenty-one cases (13 females, 8 males) were femoral neck fractures. Four cases were revision cases. Two revisions were performed due to the fact of acetabular misplacement and two with concomitant intramedullary femoral nail extraction. Two cases presented with congenital dysplastic hip (type B, Hartofylakidis et al. classification).

The mean time of the operation was 78 ± 18 min, and the length of the major incision was 6 ± 1.5 cm. The average length of stay was 1.3 days. The vast majority of patients were discharged on the first postoperative day, and all of them were discharged for home. The postoperative transfusion rate was 2% (seven cases). All patients were mobilized on the day of the operation.

Six cases presented complications: four intraoperative femur fractures and two peroneal nerve palsies. The fractures of the femur were two Vancouver B, one Vancouver C, and one Vancouver AL. The Vancouver B fractures were revised with a standard posterior approach and implantation of a Wagner-type femoral stem and cerclage wires. The Vancouver C was revised with a standard posterior approach and implantation of a Wagner-type femoral stem. The Vancouver AL fracture was treated conservatively. Regarding the peroneal palsies, drop foot was noticed immediately postoperatively, and clinical improvement was noticed after six months in both cases. No infection and no hip dislocation were noticed in any case.

## 3. Technique Modifications

While performing the SuperPATH approach, we followed exactly the steps described by Chow [13]. There have been papers that describe various aspects of the initial technique in detail such as the placement of the acetabular cup [14,15]. Quitmann [14] describes the use of a guidance tower for placing the acetabular cup, while Della Torre et al. [15] point out the importance of the transverse acetabular ligament.

We noticed that after the first 50 cases (i.e., learning curve), the primary surgeon and the whole surgical team felt more comfortable with the approach. This number of cases as a learning curve is consistent with the literature [2], as the primary surgeon and the team were familiar with both the PATH and the classic posterolateral approaches.

After some cases, and definitely after the learning curve period, we performed some modifications that made the SuperPATH more friendly to the surgeon. These modifications are described according to the corresponding steps of the SuperPATH approach as presented by Chow in 2017 [13].

**Figure 1:**
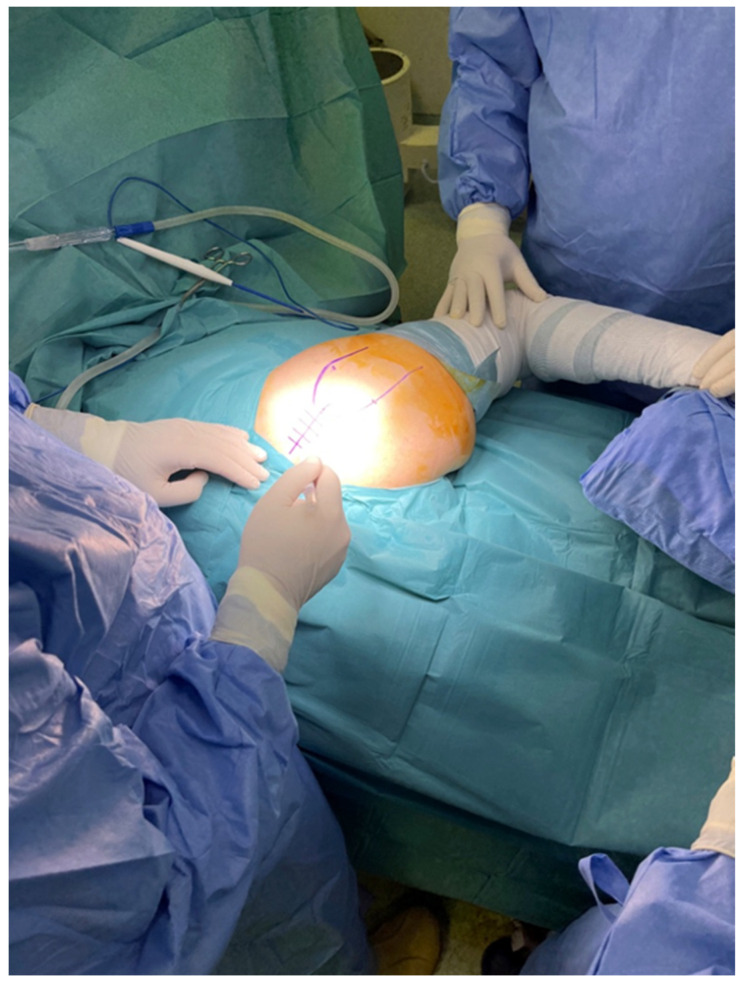
Positioning the patient as far from the surgeon as possible.

Regarding positioning the patient, we used the described standard lateral decubitus position with the involved leg in the “home position”, that is, 45° to 60° of flexion, 20° to 30° of internal rotation [13]. However, we recommend that the patient in the lateral decubitus position be placed not in the middle of the table but as far from the surgeon as possible. In this decentralized position, the available adduction will be maximized, since the distal femur and the knee will be outside the edge of the surgical bed and will not limit the adduction move (Figure 1).

**Figure 2:**
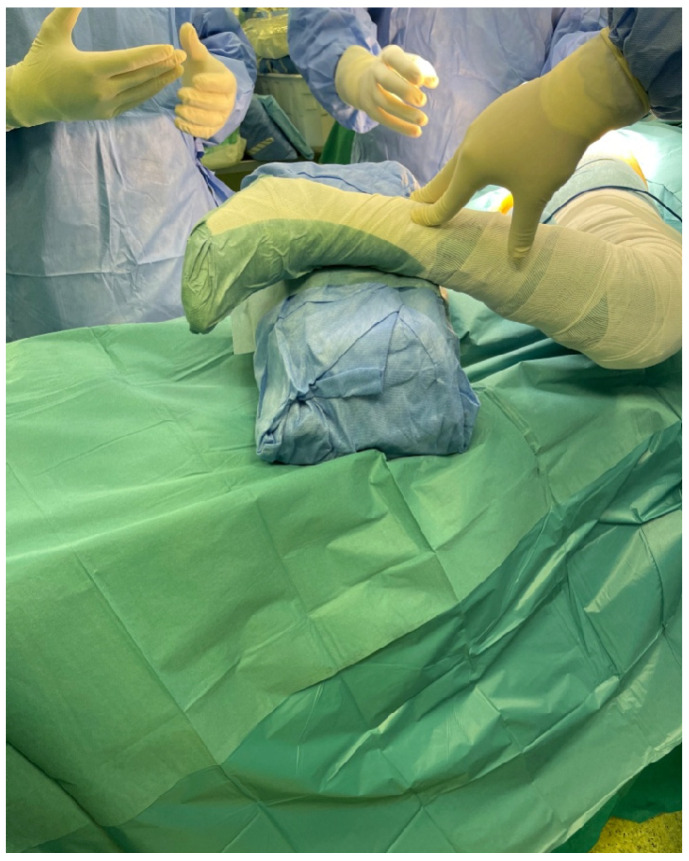
Use of a sterilized standard linen pack for elevation of the foot.

Chow recommends the use of a padded Mayo stand to elevate the foot and in that way provide a slight adduction during the operation [13]. We noticed that using a sterilized standard linen pack will provide adequate elevation of the foot and, subsequently, the needed slight adduction, and also it is much handier due to the fact of its mobility (Figure 2).

**Figure 3:**
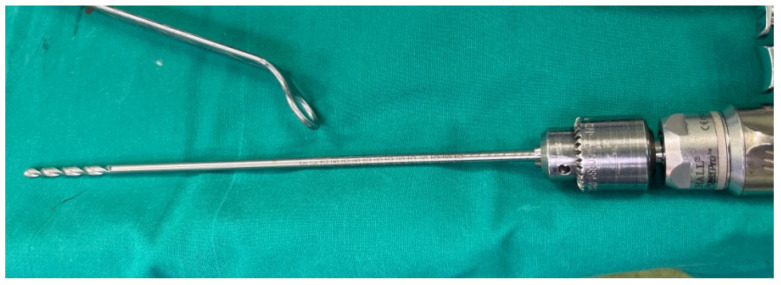
4.5 mm drill guide.

In order to create an adequate and accurate femoral channel that will be used for femoral preparation, we perform an initial intramedullary drilling. This initial drilling allows the surgeon to identify the course of the femoral canal and assess the bone density. During the first procedures, we used a 3.5 mm drill guide that was included at the instrumentation set. However, we noticed that this drill guide was rather flexible and susceptible to breakage and, thus, we replaced it with a 4.5 mm drill guide (Figure 3). In this step of the procedure, awareness must be raised in cases of patients with fragile bones in order to avoid cortical penetration.

After the neck resection, the use of two Schanz pins was described by Chow to rupture the ligamentum teres and remove the femoral head [13]. We noticed that in many cases the Schanz pins did not provide adequate grip in the femoral head due to the low bone quality of some femoral heads and the small diameter of the pins. Thus, we used a standard corkscrew to remove the femoral head.

**Figure 4:**
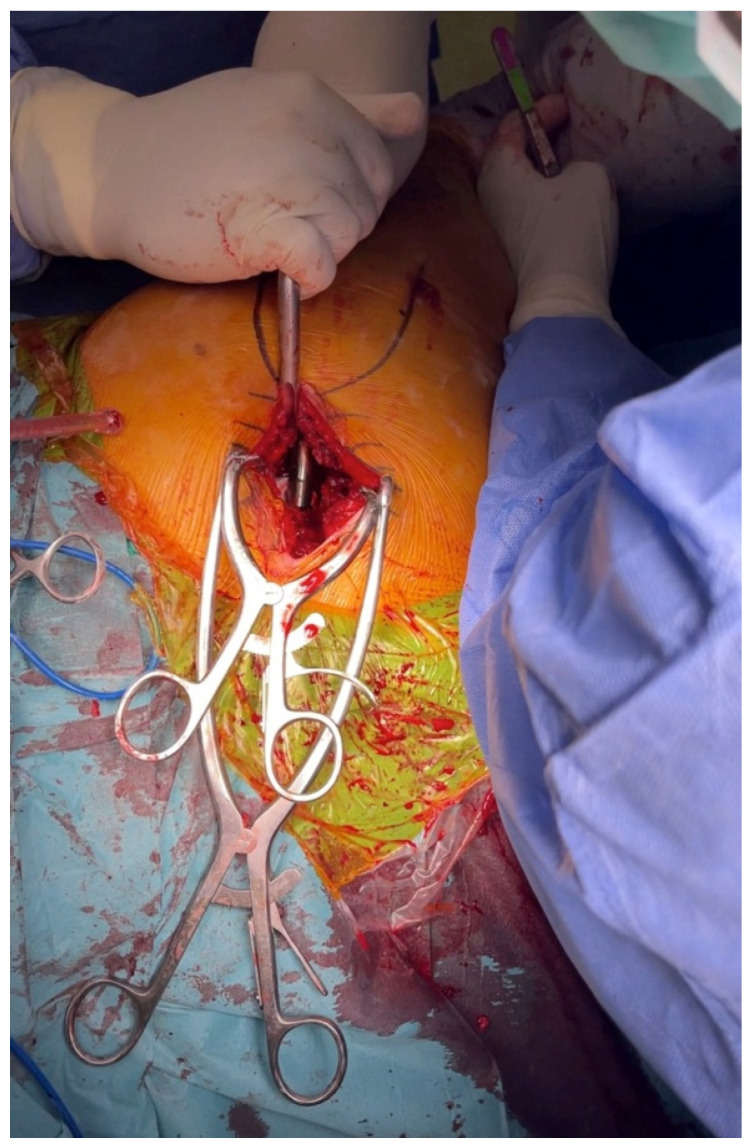
Use of a hook for reduction.

Regarding reduction, both trial and final, we used a slightly different method than the one that is described with a T-handle by Chow [13]. First, we placed the head, either the one for trial or the head of the implants, in the acetabular liner. Then we used a hook that was placed in a small circular socket located at the lateral aspect of the shoulder of the broach or the implantation stem. The hook was placed appropriately under the direct view of the surgeon. The first assistant, who was directly across, handled the hook and began a special maneuver. They elevated and slightly abducted the femur and, at the same time, pulled the hook and, subsequently, the femur by applying a steady attraction force. The surgeon, with a direct view, could see the correlation between the neck and the head and could maneuver the hip by internally or externally rotating it from the foot. When the neck was placed in the proper position, the first assistant released the attraction force, and the hip was reduced (Figure 4).

In order to achieve optimal results regarding limb length, we use intraoperative fluoroscopy in certain stages of the procedure. Firstly, when we completed the femoral broaching, we took an intraoperative X-ray. If the broach was as deep in the femur as planned preoperatively, we proceeded to the neck cut using a double-sided blade. The cut was made using the proximal end of the broach for guidance [14]. The next X-ray was taken after the trial reduction of the hip. With this X-ray we were able to assess the position of the acetabular cup and the limb length. A last intraoperative X-ray could be taken to check the final reduction before closure, if needed. However, even though intraoperative fluoroscopy has been proven very helpful during various steps of this technique, it still remains a limitation of the SuperPATH approach.

## 4. Discussion

The SuperPATH approach is a new addition in minimally invasive THA that combines the advantages of the SuperCap approach and the PATH approach [2,9,10,11,15]. The use of this approach allows for maximal tissue sparing through preservation of the external rotators and minimizing stretching of the gluteus medius. Due to the fact of this soft tissue preservation, there are no particular restrictions regarding range of motion postoperatively. The SuperPATH approach can significantly reduce the incidence of complications in patients who afterwards are able to achieve a high level of function with a very low dislocation risk. In addition, there is no need to use special traction beds and equipment. Additionally, the approach is extensile, and there is the possibility of an incision extension if needed in complex cases [15]. If there is a need for an extended view of the femur, there is always the option to release the dorsal capsule or the external rotators [14].

Although there are still no adequate long-term studies regarding the SuperPATH approach, short- and mid-term studies have shown excellent and reproducible results, with complication and readmission rates lower than those published in other studies regarding alternative total hip approaches.

Our experience with the SuperPATH approach has produced results concomitant with the current literature. Regarding hospitalization, there are studies that clearly show that the length of stay in the hospital for a patient who had hip replacement via SuperPATH was shorter compared to conventional approaches [5,6,16]. The rate of our intraoperative and perioperative complications is considered relatively low with six incidents in 344 cases (1.7%), most of which occurred during the learning curve’s early stages. The learning curve is usually used to evaluate the difficulty of a new minimally invasive technique. The shorter the learning curve, the easier to master the technique. The lead descriptive indicators include operation time, bleeding, volume, conversion rate, complication occurrence, length of hospital stay, and surgical efficacy [2]. A study in 2015 assessed early outcomes and learning curves for both PATH and SuperPATH approaches and found that the PATH group operative time reached a plateau by case 40, but the SuperPATH operative time continued to decrease by case 50 [17].

The problems that have been encountered by surgeons performing the SuperPATH approach include long operative times, difficulty retaining the external rotators via a small incision, limited intraoperative vision and operating space, and higher risk of early complications [2]. We faced some of these as well, and this is the reason we tried to improve some steps of the procedure. The modification we propose regards positioning the patient as far from the surgeon as possible; we believe this optimizes the view for the surgeon. Furthermore, by not using a Mayo table, the ability of a more mobile limb, the position of which can be adapted following the needs of the surgeon, is more helpful during the operation. With the reduction technique modification we propose, the primary surgeon can assemble the neck of the stem with the head under direct view by making some minor corrective movements, since the main force for the reduction is applied by the first assistant via the hook. Of course, adequate muscle relaxation is of major significance, and impeccable cooperation with the anesthesiologist is crucial in this stage of the procedure. If the reduction is not achievable, a partial release of the piriformis tendon might be required. In addition, the use of intraoperative fluoroscopy can provide much information regarding limb length and help the surgeon choose the right size of the implants to achieve the best results. Moreover, if there is any suspicion of an intraoperative fracture, this could be detected in time and treated appropriately.

Quitmann supports that the SuperPATH approach is beneficial for obese patients, as there is not necessarily a need for a very long incision because the procedure is performed in a “slot” with a minimal dissection of subcutaneous tissue [14]. We find this suggestion to be viable, as we have performed the SuperPATH approach successfully in 88 obese patients. Especially with the modifications we propose regarding the positioning of the patient and the reduction technique, these particular steps of the procedure become easier.

Furthermore, the wider range of motion that is provided by positioning the patient closer to the edge of the table, might provide some more adduction and internal rotation in patients with a very stiff hip. In addition, a slight anterior tilting of the pelvis, which is allowed since the knee and the distal part of the thigh are outside the table, might provide a better view for the approach.

Another application of the SuperPATH approach is the treatment of femoral neck fractures with either a total hip replacement or a hemiarthroplasty [14,18]. We have performed the SuperPATH approach in 48 patients with a femoral neck fracture. In 25 of them a total hip replacement was performed, and 23 patients were treated with a hemiarthroplasty. In all cases the femoral head was left in situ until preparation of the femur was completed. There have been no intraoperative or perioperative complications regarding these patients.

There have been developments in minimally invasive surgery, and more surgeons are becoming familiar with these new and improved techniques. These techniques have found their way to the center of clinical research and many studies are under development. Regarding the SuperPATH approach, there are studies that show promising results and suggest that its short-term outcomes in hip replacement are better compared to conventional approaches despite the limited visualization that accompanies a small incision [5]. The SuperPATH approach showed better results in decreasing incision length and early pain intensity as well as improvements in short-term functional outcome [2].

## 5. Conclusions

In general, for surgeons who are familiar with the standard posterolateral approach, the SuperPATH approach is a reliable and safe method with promising results for the patient. In order to improve the surgical effect and facilitate some steps in the procedure, we shared our experience and recommend some modifications that might seem helpful for surgeons who wish to perform or optimize the SuperPATH approach. Certainly, there is a need for larger prospective research in the future to investigate the outcomes of the SuperPATH technique and to compare them with other approaches.
